# Supplementary material for: Bioactive Properties of Microencapsulated Anthocyanins from Vaccinium floribundum and Rubus glaucus
Source: Molecules. 2024 Nov 21;29(23):5504. doi: 10.3390/molecules29235504 (PMC11643421; doi:10.3390/molecules29235504)
Supplement: Supplementary file 1 [file molecules-29-05504-s001.zip › molecules-3264987-supplementary.pdf]

Figure S1: FTIR Spectral Curves of Anthocyanins from *Vaccinium floribundum*. A) FTIR spectrum of non-encapsulated anthocyanins from *V. floribundum.* The spectrum displays the characteristic absorption bands corresponding to the functional groups present in the anthocyanins, indicating the molecular structure of the non-encapsulated form. B) FTIR spectrum of microencapsulated anthocyanins from *V. floribundum*. The encapsulation process alters the spectral profile, suggesting changes in molecular interactions or conformations due to the encapsulation matrix.

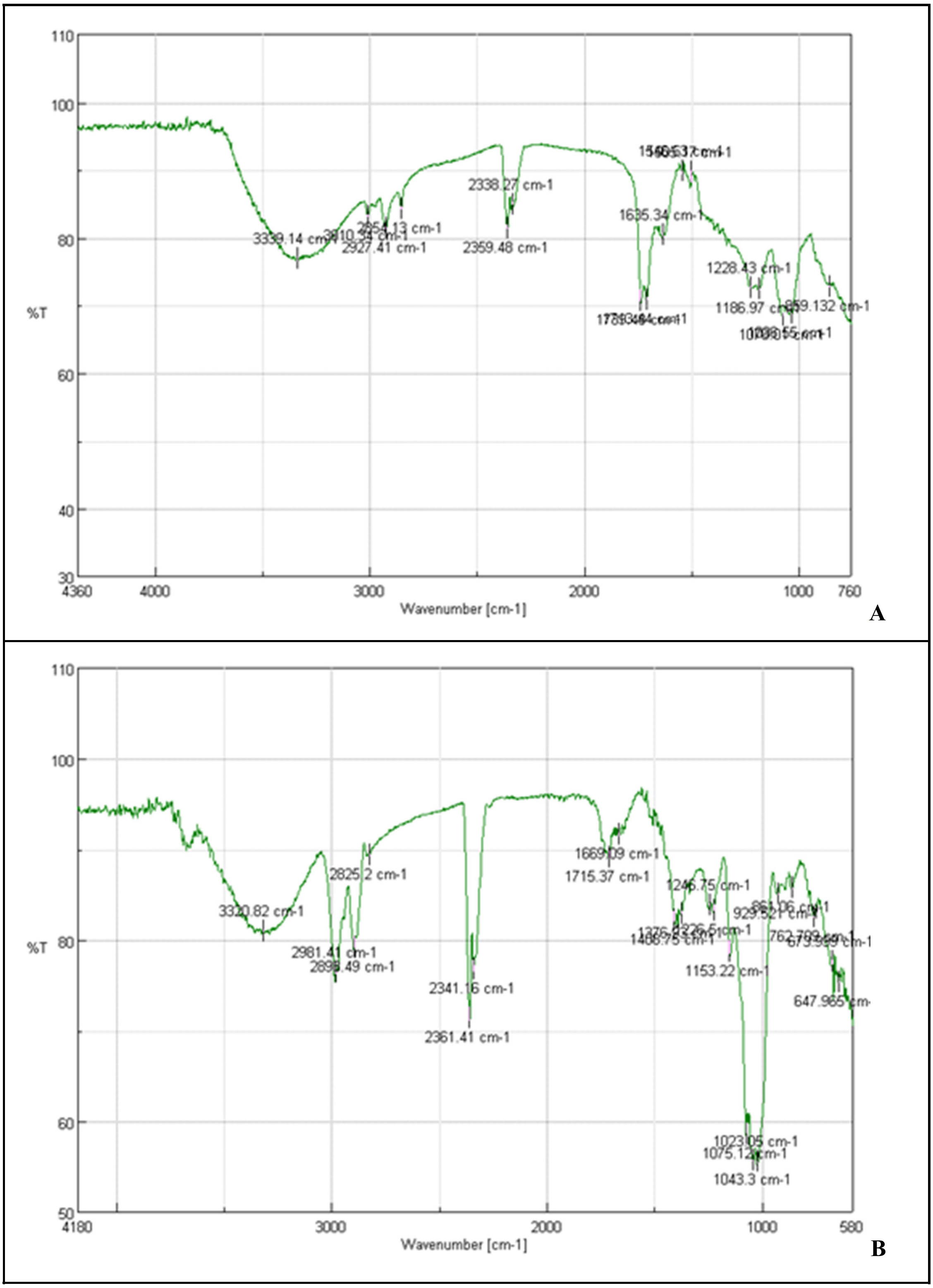

Figure S2: FTIR Spectral Curves of Anthocyanins from *Rubus glaucus*. A) FTIR spectrum of non-encapsulated anthocyanins from *R. glaucus*. This spectrum highlights the absorption bands associated with the functional groups of anthocyanins in their free form, offering insight into their chemical structure. B) FTIR spectrum of microencapsulated anthocyanins from *R. glaucus*. The microencapsulation introduces spectral changes that may reflect interactions or modifications of the anthocyanin molecules within the encapsulation matrix.

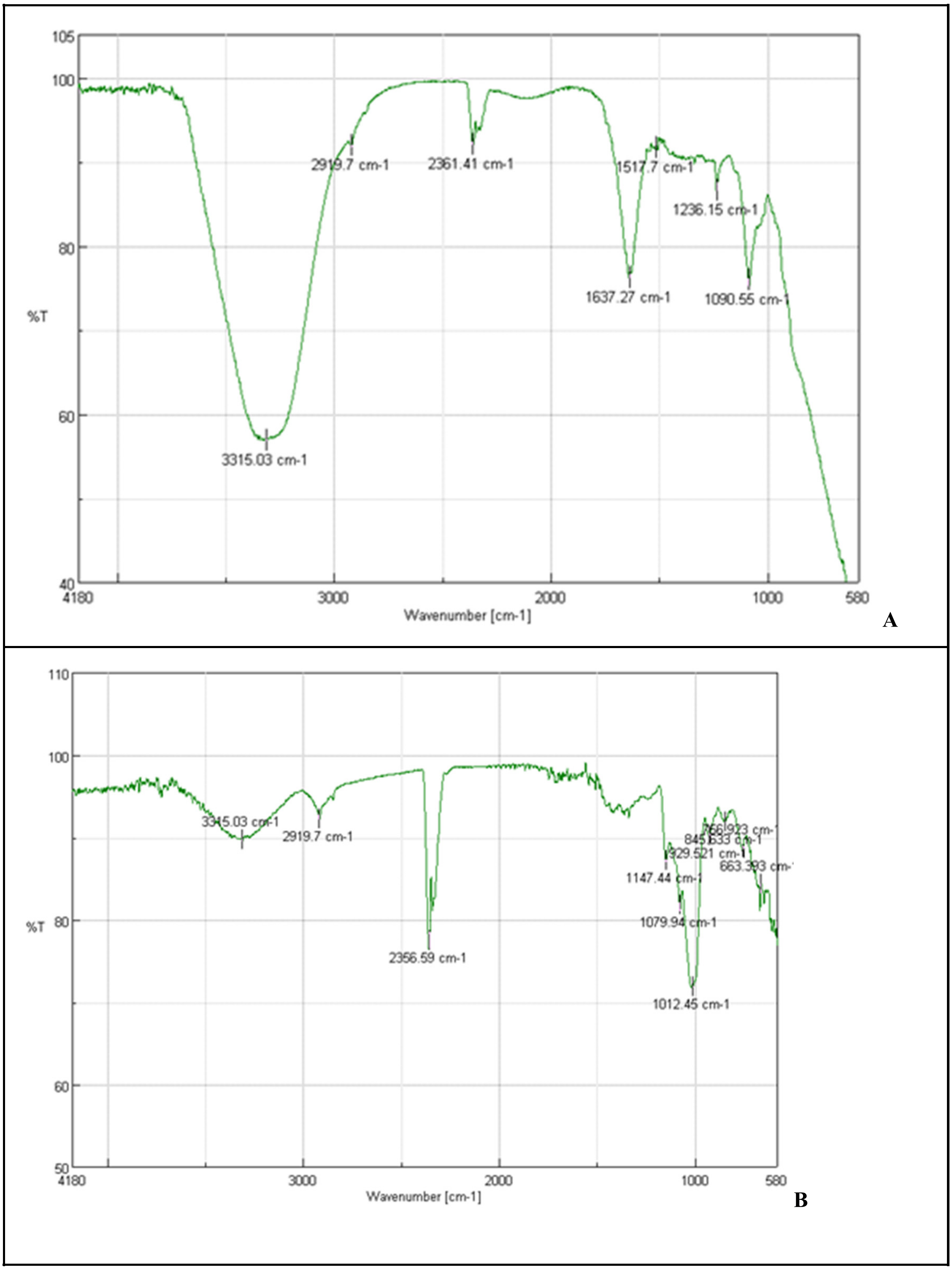

Figure S3. Statistical analysis between IC_{50} values from *R. glaucus* vs. *V. floribundum* microencapsulations (unpaired t-test, $p < 0.001$).

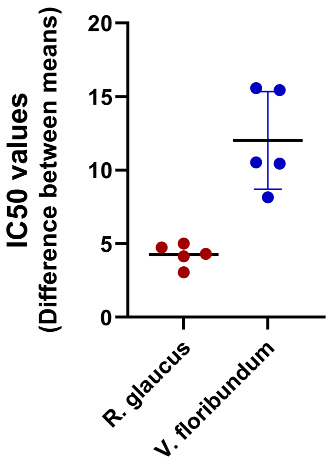

Table S1. Corresponding anthocyanin concentrations (µg/mL) in microencapsulated extracts at the IC_{50} values for tumor and non-tumor cell lines after 72 hours.

Cell lines	MDAMB231	SKMEL103	HCT116	HT29	NIH3T3
R. glaucus	5.80 ± 0.35	4.29 ± 0.84	7.04 ± 0.13	6.66 ± 0.28	6.04 ± 0.51
V. floribundum	9.78 ± 2.00	18.55 ± 1.21	18.70 ± 4.26	12.63 ± 2.64	12.52 ± 1.88

Table S2. Inhibitory concentration values (IC_{20}) of maltodextrin against tumor and non-tumor cell lines at 72 h. IC_{20} values are crucial in assessing safety and toxicity, particularly when minimizing harm to normal cells is essential.

Cell lines	MDA-MB-231	SKMEL-103	HCT116	HT29	NIH3T3	RAW264.7
**mg/mL**	52.16 ± 0.78	38.46 ± 1.9	36.26 ± 0.58	24.99 ± 1.14	13.17 ± 1.63	9.11 ± 1.63
